# Newly-found channels in the interatrial septum of the heart by dissection, histologic evaluation, and three-dimensional microcomputed tomography

**DOI:** 10.1371/journal.pone.0246585

**Published:** 2021-02-08

**Authors:** Mi-Sun Hur, Seunggyu Lee, Chang-Seok Oh, Yeon Hyeon Choe

**Affiliations:** 1 Department of Anatomy, Catholic Kwandong University College of Medicine, Gangneung, Korea; 2 Division of Applied Mathematical Sciences, Korea University, Sejong, Korea; 3 Department of Anatomy and Cell Biology, Sungkyunkwan University School of Medicine, Suwon, Korea; 4 Department of Radiology, Samsung Medical Center, Sungkyunkwan University School of Medicine, Seoul, Korea; Jagiellonian University Medical College, POLAND

## Abstract

A minute thrombus can pass through a small type of interatrial communication, which can result in a stroke or transient ischemic attack and several associated symptoms. This study sought to investigate a new type of interatrial communication. Thirty-one hearts from embalmed adult cadavers were investigated. Each interatrial channels (IACs) was classified as either an open or obstructed channel according to the connection of each hole on the right and left surfaces of the interatrial septum. Open channels were found in two specimens (6.5%). Both open and obstructed IACs followed tortuous courses through the interatrial septum. On the right surface of the interatrial septum, the hole was usually found adjacent to the left border of the interatrial septum between the opening of the superior vena cava into the right atrium and the superior margin of the fossa ovalis. Conversely, holes on the left surface of the interatrial septum were usually found in the upper and middle parts adjacent to the left border of the interatrial septum. This novel finding is expected to support our understanding of the onset of possible symptoms such as stroke in the absence of classical atrial septal defects.

## Introduction

Interatrial communications usually involve an atrial septal defect (ASD) and a patent foramen ovale (PFO) [1]. An ASD is a direct communication between the atrial cavities that facilitates the shunting of blood [2], while a PFO is a small channel that has hemodynamic consequences as a remnant of the fetal foramen ovale [3].

The present study investigated the interatrial channel (IAC), which appears to be a new type of interatrial communication that has not been reported previously. ASDs and PFOs exhibit different incidence rates, sites, morphology, and features depending on their respective types. For example, ASDs have been classified into four types according to their anatomical locations in the interatrial septum. Ostium secundum ASDs are the most common, approximately 75% of all ASDs. They result from a defect of the septum primum and are the most amenable to percutaneous device closure. Ostium primum ASDs involve the atrioventricular valves are, therefore, also called partial atrioventricular defects. Superior or inferior sinus venosus ASDs are commonly associated with anomalous pulmonary vein returns. And the rarest of ASDs is coronary sinus that drains from the coronary sinus os into the right atrium [4]. Accurate identification of the type of interatrial communication is necessary for their evaluation and for selecting the most appropriate device to achieve their complete closure.

While ASDs and PFOs represent different types of interatrial communication, they both have an increased risk of paradoxical embolism leading to cryptogenic stroke [5]. Cryptogenic (or unexplained) stroke is present in 30–40% of ischemic stroke patients [6]. A paradoxical embolism or transient ischemic attack can occasionally be the first clue suggesting the presence of an ASD. In one study, a patient (a pregnant woman) with an ASD was predisposed to paradoxical embolism due to a hypercoagulable state and enhanced right-to-left shunting arising from an increased plasma volume and a decreased peripheral vascular resistance [5]. PFO is more prevalent in patients with cryptogenic stroke than in patients with a stroke of a known cause [7], and the presence of a PFO is strongly associated with neurological events such as cryptogenic ischemic stroke in young patients [8]. A blood clot from the venous circulatory system is able to pass from the right atrium directly into the left atrium via a PFO, rather than being filtered by the lungs, and thereupon into systemic circulation toward the brain, where it causes an ischemic stroke [9,10]. Right-to-left shunting may occur at rest or only transiently with an increase in right-sided pressure due to coughing [11].

Akhondi et al. (2010) [12] reported that smaller PFO size without the presence of atrial septal aneurysm may still be associated with significant strokes and there is no significant correlation between stroke volume on magnetic resonance imaging and PFO size on cardiac echocardiography. Also they described that it is possible to have a large stroke with a small PFO and PFO size and morphology should not be used as the only criteria for whether a PFO should be closed. Thus, although the IAC is a minute communication through the interatrial septum, it can be considered regardless of its size.

The atrial septal pouch is an anatomic variant of the interatrial septum. The atrial septal pouch was defined as a small, kangaroo pouch–like structure located on the human interatrial septum [13,14]. Krishnan and Salazar (2010) [15] reported that incomplete fusion of the septum primum and septum secundum results in a pouch that, in the majority of instances, communicates with the left atrium cavity. The morphology of the left sided septal pouch may favor blood stasis and predispose to thromboembolic events [16]. Hołda and Koziej (2018) [14] demonstrated meta-analysis of association between left-sided atrial septal pouch and cryptogenic stroke.

Small emboli that shed from small varicose veins or hemorrhoids might not be detectable using current imaging modalities [5]. Residual shunting after ASD closure might result in persistent volume loading of the right heart and pulmonary circulation [17]. Windecker et al. (2000) [18] found that the presence of a postprocedural shunt of the PFO was a predictor of recurrent thromboembolic events. These observations imply that a minute thrombus might pass through a small type of interatrial communication, resulting in a stroke or transient ischemic attack and several associated symptoms.

The purpose of this study was to investigate a new type of interatrial communication. The findings are expected to be helpful for understanding the nature of symptoms such as stroke in the absence of classical ASDs or PFOs. Moreover, the obtained data will also provide helpful information for detecting.

## Materials and methods

### Specimens and dissection analysis

Thirty-one hearts from embalmed Korean adult cadavers (17 males and 14 females; range, 40–89 years) were investigated. Before dissection, the external structure of heart was carefully checked. The specimens used in this study had no external injuries resulting from a trauma, interventional or surgical procedures. The IACs were dissected under a surgical microscope. The presence and sites of holes on the right and left surfaces of the interatrial septum were identified, and the largest and smallest diameters of each identified hole were measured using digital calipers. To trace IACs that began from holes, the endocardium adjacent to the holes was removed. Cardiac muscle and soft tissues surrounding the holes were then cut in the direction of the IAC under a surgical microscope. Next, the IAC was traced to determine whether it was open or obstructed as it passed through the interatrial septum. After tracing the IAC by dissection, a thread was passed through it to check whether two holes were connected and to measure the length of the IAC.

### Histological analysis

Histological sectioning was performed on one specimen from thirty-one hearts. A formalin fixed and paraffin embedded tissue was prepared to obtain transverse sectional images of the IAC passing through the interatrial septum. The 5-μm-thick transverse section was stained with hematoxylin and eosin and the IAC and its adjacent structures were examined microscopically to elucidate their compositions.

### Micro-computed tomography (MICRO-CT) analysis

Eight from the thirty-one hearts were scanned using micro-CT (Inveon Micro-PET/CT scanner; Siemens Medical Solutions, Knoxville, TN, USA). Specimens were scanned with a 1.5-mm-thick aluminum filter using the following settings: 720 rotation steps spanning 360° in a constant step size of 0.5°, source settings of 70 kV and 400 μA, and exposure time of 800 ms per step. After applying 2 × 2 pixel binning, the effective pixel size (or resolution) was approximately 20.3 μm. For each scan, the dataset was reconstructed with a down-sampling factor of 2 using the Inveon Acquisition Workplace software package (Siemens Medical Solutions) and by implementing the modified Feldkamp filtered back-projection algorithm (Shepp–Logan filter). The reconstructed images were then imported using the Inveon Research Workplace into the accompanying two-dimensional and three-dimensional (3D) biomedical image analysis software package (CT Bone Visualization and Analysis; Siemens Medical Solutions) for visualization and analysis. When performing micro-CT, a radiocontrast agent (iomeprol; Iomeron 400 mgI/mL, Bracco SpA, Milan, Italy) was applied adjacent to the hole that seems to be connected to the holes on the opposite surfaces of the interatrial septum.

Micro-CT data in Digital Imaging and Communications in Medicine format with a slice thickness of 0.107 mm were transferred to the Mimics software (version 21.0; Materialise, Leuven, Belgium) on a personal computer. On each sectional axial, coronal, and sagittal image, the IAC and interatrial septum were identified and colored pink and yellow, respectively. The 3D images of the IAC passing through the interatrial septum were reconstructed into 3D models and the software was subsequently used to render the 3D models in a semitransparent manner to allow visualization of the entire channel passing through the interatrial septum.

### Computed Tomography (CT) analysis

CT was performed using a third-generation dual-source CT scanner (Somatom Force, Siemens Healthineers, Germany). Spatial resolution in CT was 0.46 mm x 0.46 mm x 0.5 mm.

### Ethical approval

All cadavers had been legally donated to Catholic Kwandong University College of Medicine. The present study was conducted in accordance with the Declaration of Helsinki. None of the transplant donors were from a vulnerable population and all donors or next of kin provided written informed consent that was freely given. This study was approved by the institutional review board of the Samsung Medical Center (IRB ID No. NON2020-005).

## Results

### Presence and locations of the tiny holes on the surfaces of the interatrial septum

IACs were observed to open into the atrial cavities thorough a hole on both sides or one side of the interatrial septum. Several tiny holes of various sizes and shapes were found on the surfaces of the interatrial septum. The holes were found on both the right and left surfaces of the interatrial septum in 22 of the 31 specimens (71.0%). The holes on the right surface were usually oval, while those on the left surface were often either linear or round. Holes were observed only on one side of the right and left surfaces of the septum in seven of the 31 specimens (22.6%), while no hole was found on any surface in two of the specimens (6.5%).

**Fig 1:**
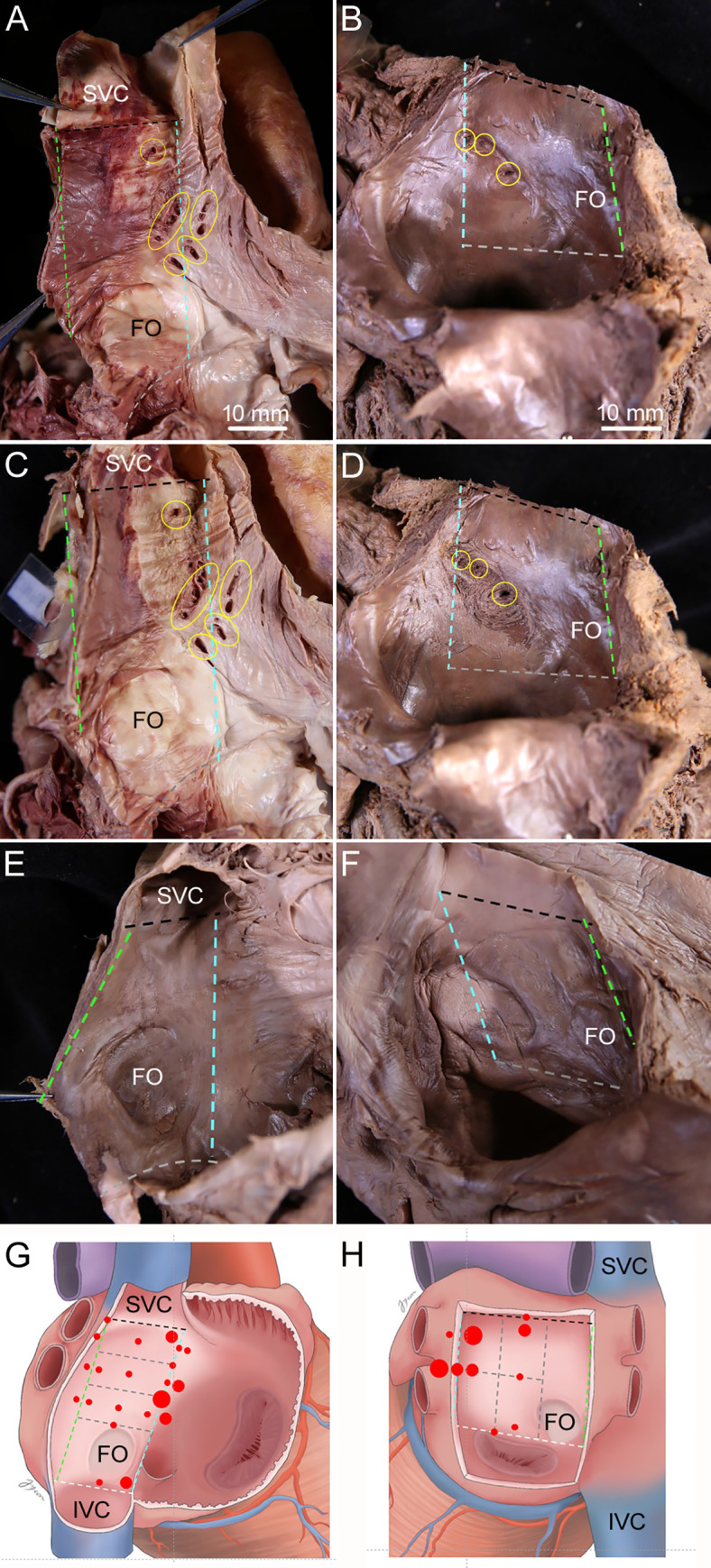
Sites of tiny holes on the surfaces of the interatrial septum. Dissection image (A, C, and E) and a diagram (G) showing where the holes on the right surface of the interatrial septum were usually. Found: adjacent to the left border of the interatrial septum at one-third of the distance from the margin of opening of the superior vena cava (SVC) into the right atrium to the superior margin of the fossa ovalis (FO). Dissection image (B, D, and F) and a diagram (H) showing where the holes on the left surface of the interatrial septum were usually found: in the upper and middle parts of interatrial septum adjacent to the left border. The endocardium adjacent to the holes that seems to be connected to the holes on the opposite surfaces of the interatrial septum was removed to trace IACs that began from holes on the right surface (C) and the left surface (D) of the interatrial septum. Specimen had no holes on the right surface (E) and the left surface (F) of the interatrial septum. Dashed lines indicate the borders of the interatrial septum, with each border indicated using the same color. Yellow circles indicate holes on the surfaces of the interatrial septum. Red dots and their size indicate the sites and relative prevalence of the holes, respectively, with the large, mid-sized, and small red dots representing more than 10, five to 10, and less than five cases, respectively. The right and left atria were opened to show holes on the surfaces of the interatrial septum. IVC, inferior vena cava. Scale bar 10 mm.

Holes on the right surface of the interatrial septum were usually found adjacent to the left border of the interatrial septum at one-third of the distance from the margin of opening of the superior vena cava into the right atrium to the superior margin of the fossa ovalis. Holes on the left surface were usually found in the upper and middle parts of the interatrial septum adjacent to the left border of the septum (Fig 1).

### Classification of the IAC by dissection and micro-CT images

**Fig 2:**
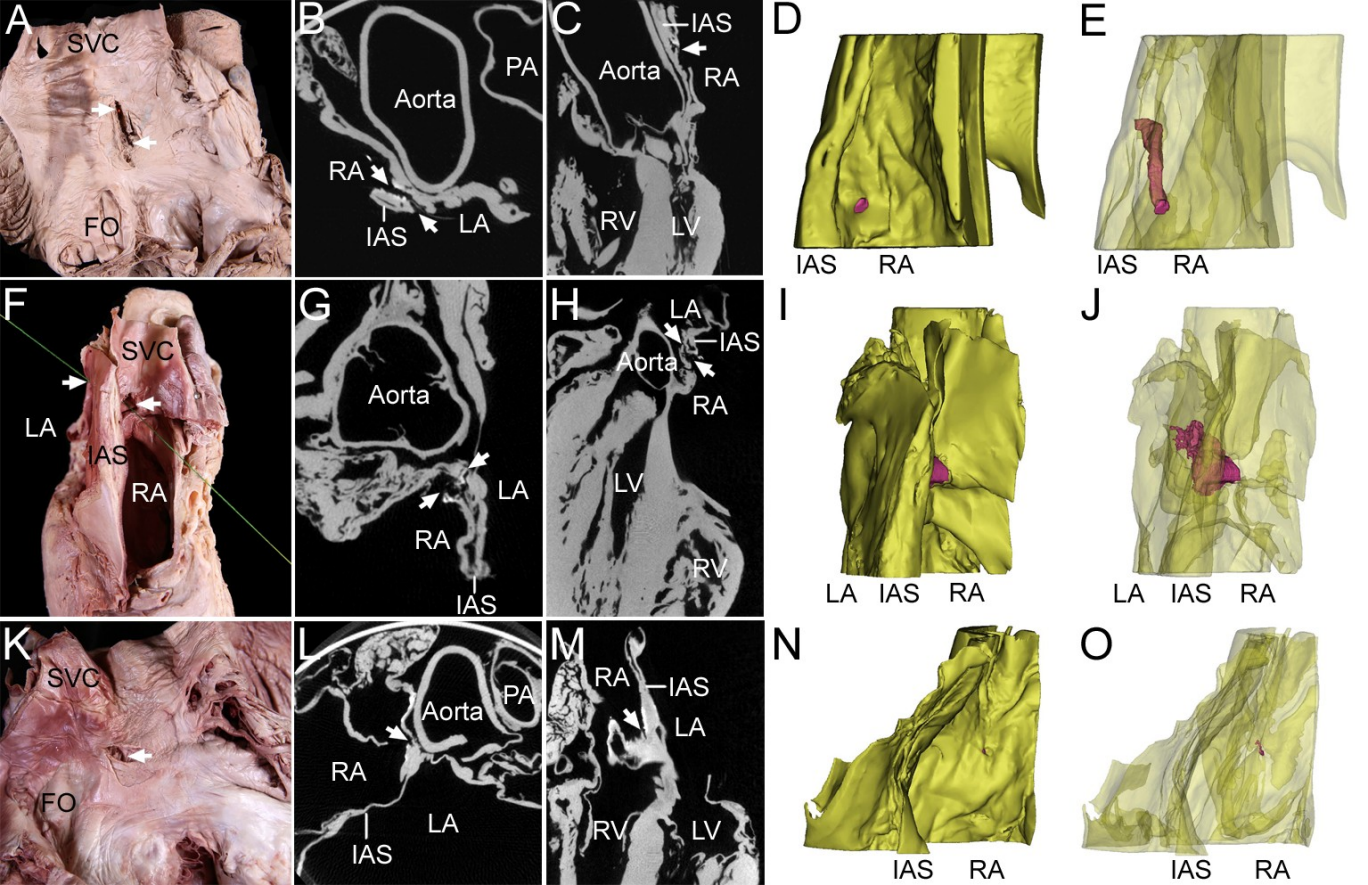
Classification of the IAC in dissection images, micro-CT Images, and 3D models. In two specimens of an open channel (one specimen for A-E, the other specimen for F-J), the holes (arrows) on the two sides were found to be connected to each other via a tortuous channel in dissection images (A, F), axial-plane micro-CT images (B, G), sagittal-plane micro-CT images (C, H), and 3D models (D, E, I, and J). In a specimen of an obstructed channel (one specimen for K-O), holes (arrows) on the two sides were not connected in a dissection image (K), axial-plane micro-CT image (l), sagittal-plane micro-CT image (M), or 3D model (N, O). In the 3D model, the yellow structure is the interatrial septum (IAS) and the red structure is the IAC. The model was rendered semitransparently so that the entire channel passing through the IAS could be visualized. The same specimen was used for the dissection image, micro-CT images, and 3D model. For the specimen in panel A, light was allowed to shine through the open channel from the connected hole on the opposite surface. For the specimen in panel F, a thread was passed through the IAC to show the connection of each hole. The right atrium (RA) was opened. FO, fossa ovalis; LA, left atrium; PA, pulmonary artery; SVC, superior vena cava.

Each IAC was classified as either an open or obstructed channel according to the connection of each hole on the right and left surfaces of the interatrial septum (Fig 2). Open channels were found in two specimens (6.5%, Table 1). Holes on both sides connected to each other via an open channel were found in their most common site of the present study. In cases where the holes on both sides were not connected or where holes were observed only on one side, the holes showed obstructed channels of various lengths. Both open and obstructed IACs followed tortuous courses through the interatrial septum.

**Table 1:** Size of holes and channel length of the specimens having the open type IAC (unit: mm).

Specimen Number	Sex	Age	Number of holes		Size of holes (largest x smallest diameters)		Length of open channel
			Right surface of interatrial septum	Left surface of interatrial septum	Right surface of interatrial septum	Left surface of interatrial septum	
1	Male	40	2	5	6.0 x 4.3 (hole of IAC)	0.7 (hole of IAC, linear shape)	14.9
					2.4 x 0.5	0.7 x 0.1 0.8 x 0.1 0.6 x 0.1 0.4 x 0.1	
2	Female	65	2	2	1.7 x 0.4 (hole of IAC)	0.5 x 0.2 (hole of IAC)	16.1
					0.9 x 0.4	0.2 x 0.1	

In one specimen of an open IAC, the largest and smallest diameters of the connected hole on the right septal surface were 6.0 mm and 4.3 mm, respectively, and there were several tiny muscular trabeculations within the channel. The connected hole on the left septal surface had a linear shape with a diameter of 0.7 mm, while the length of the channel was 14.9 mm. In another specimen of an open IAC, the largest and smallest diameters of the connected hole on the right septal surface were 1.7 mm and 0.4 mm, respectively, while those for the hole on the left septal surface were 0.5 mm and 0.2 mm. The length of this channel was 16.1 mm.

The micro-CT images and their reconstructed 3D models showed that the open IACs were observed to pass through the interatrial septum following tortuous courses, having irregular size and nonuniform cross-sectional shape. Conversely, the micro-CT images and their reconstructed 3D models showed that the obstructed IACs had a tortuous course and were blocked (Fig 2).

### Histological analysis of the IAC passing through the interatrial septum

**Fig 3:**
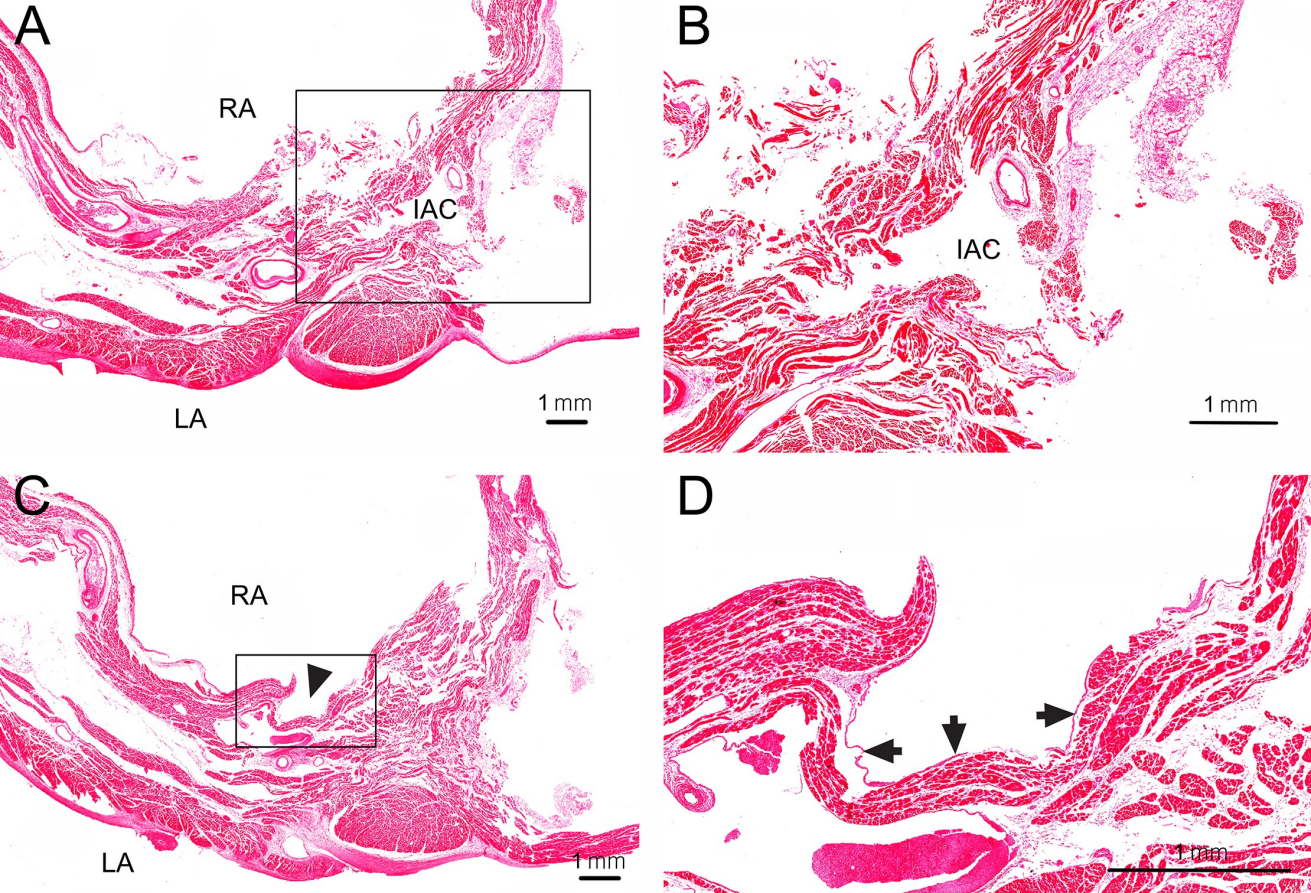
Transverse histological sections at the middle and lower levels of the open IAC (the same specimen as Fig 2A–2E) passing through the interatrial septum. (A) At the middle level of the open IAC, the interatrial septum could be microscopically distinguished into two parts: one able to be separated into two components and an inseparable part. The separable part was near the right border of the septum, while the inseparable part was near its left border. The IAC was always observed at the inseparable part. (B) Enlarged view of the rectangular inset in A. The wall of the IAC involved muscular tissues running in different directions. Endothelium was not observed in the wall of the IAC. LA, left atrium; RA, right atrium. (C) At the lower level of the open IAC, a hole (arrowhead) of the open IAC was observed on the right surface of the interatrial septum. (D) Enlarged view of the rectangular inset in C. Endocardium (arrows) was observed adjacent to the hole of the IAC.

Transverse histological sections were obtained at the middle and lower levels of an open IAC (the same specimen as Fig 2A–2E) passing through the interatrial septum and stained with hematoxylin and eosin (Fig 3). The interatrial septum could be microscopically distinguished into two parts: a part able to be separated into two components and an inseparable part. The separable part was near the right border of septum, while the inseparable part was near its left border. The IAC was always observed at the inseparable part, and the muscular tissues in its wall ran in different directions. Endocardium was observed adjacent to the hole of the IAC, and endothelium was not observed in the wall of the IAC.

### Computed Tomography (CT) of the IAC

**Fig 4:**
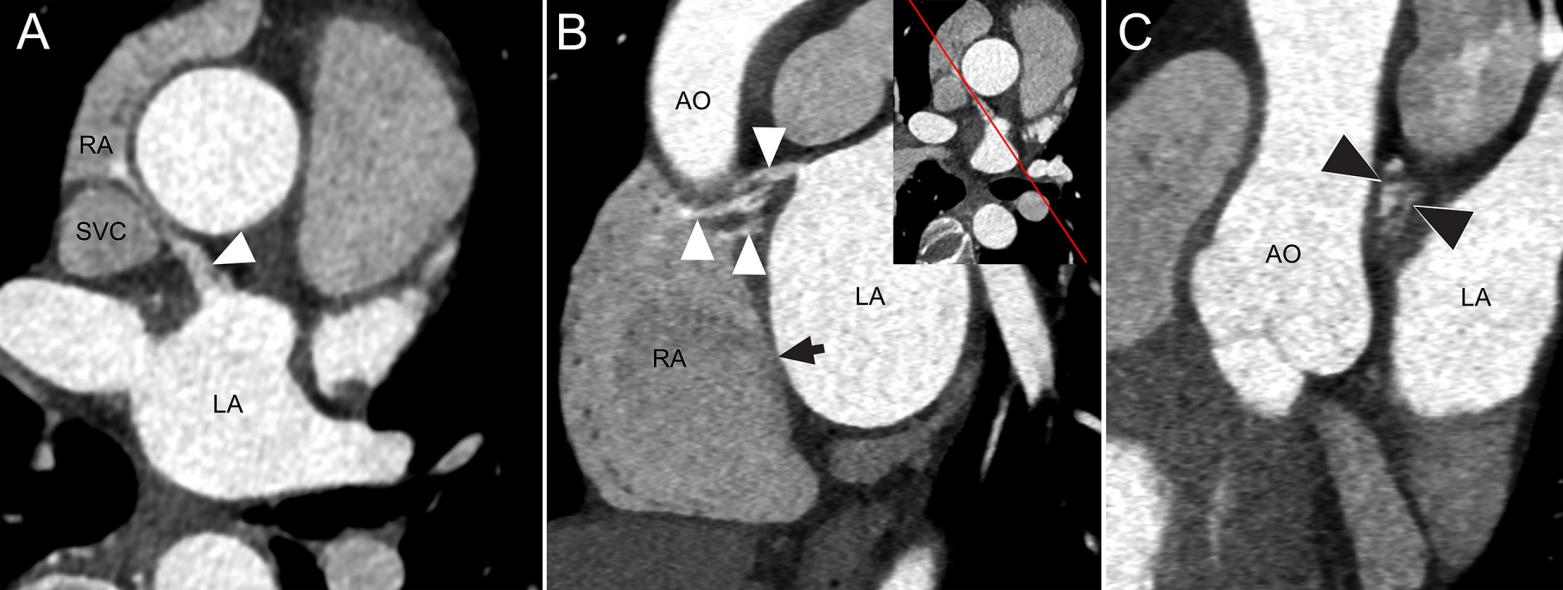
CT Images of an IAC in a 61-year-old female with Marfan syndrome. (A) Axial CT image shows a communication channel (arrowhead) between the left atrium (LA) and right atrium (RA) near the junction of the RA and superior vena cava (SVC). (B) Oblique sagittal reformatted CT image shows openings of multiple channels on the right atrial side and one channel on the left atrial side with varying thickness of the channels (2.4–4.3 mm) and length (16.5–20.8 mm). Insert image shows the direction of sagittal reformation. High attenuation of contrast-enhanced blood in the communication channels (arrowheads) indicates blood shunt from LA to RA. An arrow represents the fossa ovalis. (C) Oblique coronal reformatted image shows multiple small channels (arrowheads) in cross-section. AO, ascending aorta.

CT revealed a thin open IAC following a tortuous course through the interatrial septum of the heart of a 61-year-old female with Marfan syndrome. Stroke and other symptoms were not observed in this patient (Fig 4).

## Discussion

This study revealed the IAC—suggesting a new type of interatrial communication—by dissection, micro-CT imaging, and clinical CT images. The IAC presents the following specific features:

1. The IAC followed a tortuous course through the interatrial septum and the channel was open in 6.5% of the specimens.
2. The sites of the holes at both ends of the IAC on the surfaces of the interatrial septum differed from those of ASDs and PFOs.
3. The IAC in CT images between the left and right atria, which could cause thromboembolic events that lead to cryptogenic stroke.

**Fig 5:**
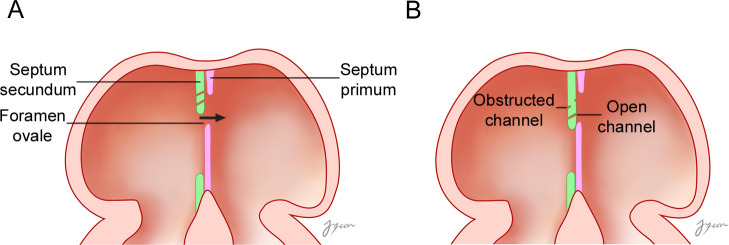
An embryological model of the formation of an IAC during the development of the atrial septa. (A) As the septum secundum develops, the blood flows from the right atrium to the left atrium, which results in the formation of several tiny channels through the septum secundum. (B) As development proceeds, most of these tiny holes became blocked completely due to the proliferation of muscle cells in the interatrial septum, with or without leaving holes on the surfaces of the septum. The blocked channel, having holes on the septal surface, evolves into a blocked IAC. Some channels were not blocked, kept open by the blood flow, and became open IACs. The arrow indicates the direction of blood flow.

The formation of an IAC can be understood by considering the embryologic development of the interatrial septum. Based on the results of the present study, a model for the formation of the IAC during the development of the atrial septa can be suggested (Fig 5). As the septum secundum develops, the blood flows from the right atrium to the left atrium, which results in the formation of several tiny channels through the septum secundum. This might be correlated with the finding of that IACs were always observed at the inseparable part of the interatrial septum. As the development proceeds, most of these channels became blocked completely due to the proliferation of muscle cells in the interatrial septum, with or without leaving holes on the surfaces of the septum. The blocked channel, having a hole on the septal surface, came to be a blocked IAC. Some channels were not blocked but instead kept open by the blood flow, remaining as open IACs. The observed tortuous course of the IAC may be due to the blood flow against the resistance of the proliferated muscular tissue in the septum. In open IACs, the holes on the right septal surface were larger than those on the left surface, which implies that blood entered the hole on right septal surface and exited from the hole on the left surface with a reduced volume and velocity due to the obstacle of proliferated muscular tissue in the middle between two holes.

**Fig 6:**
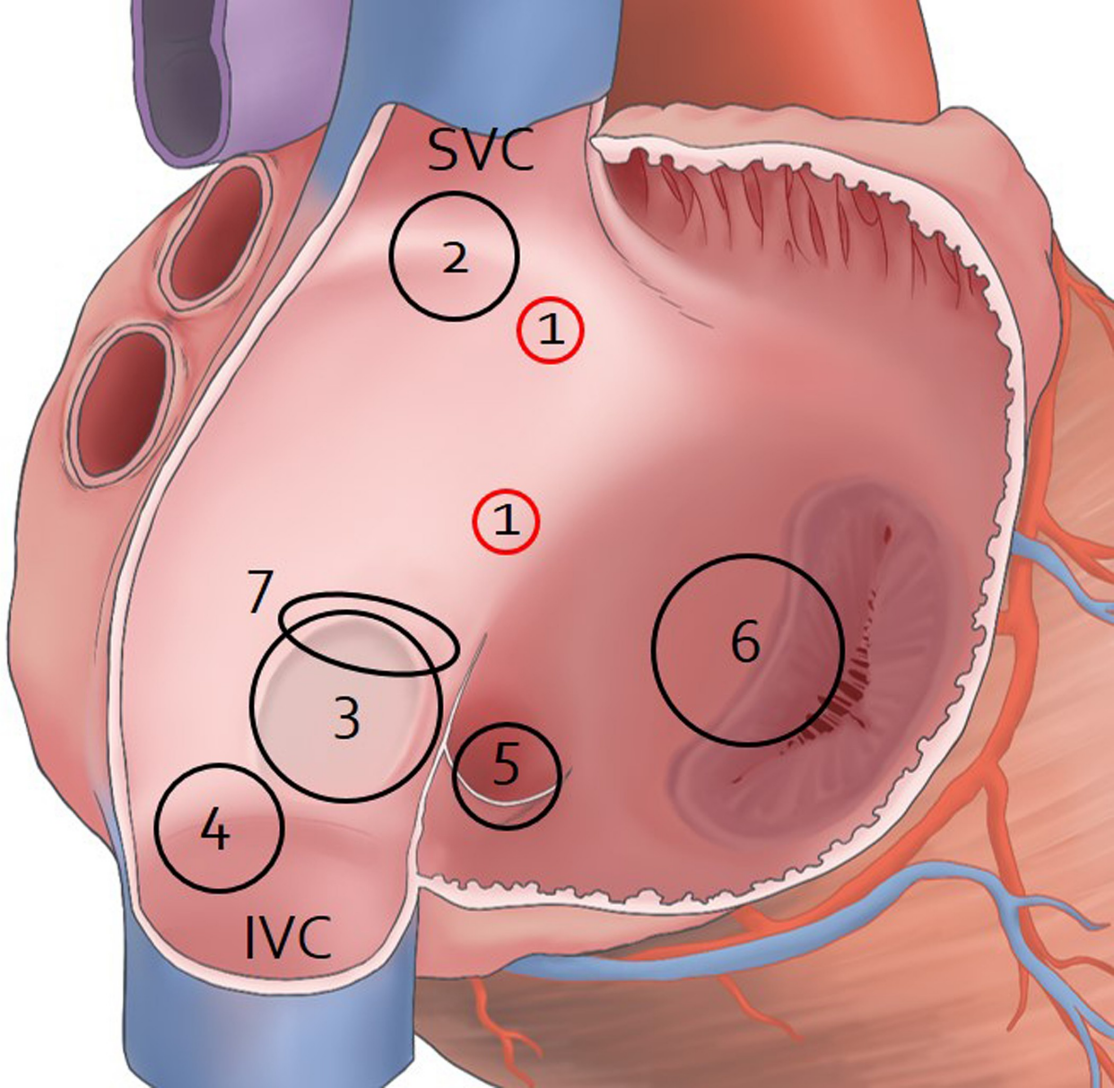
Sites of IAC, atrial ASDs, and PFO on the right surface of the interatrial septum. The right atrium was opened to show holes on the surfaces of the interatrial septum. 1, IAC; 2, Superior sinus venosus ASD; 3, Ostium secundum ASD; 4, Inferior sinus venosus ASD; 5, coronary sinus ASD; 6, Ostium primum ASD; 7, PFO; SVC, superior vena cava; IVC, inferior vena cava.

The characteristics of IACs most different from ASDs and PFOs were the site and course (Table 2 and Fig 6) [2,4,5,11,15,19–23]. The IAC is the only type of interatrial communication located above the fossa ovalis on the surface of the interatrial septum. Meanwhile, the most common type of ASDs are holes known as “secundum defects” that occur in the fossa ovalis. Other defects such as in the superior and inferior sinus venosus, coronary sinus, and ostium primum lie outside the area of the true septum [2,24]. The PFO is a tunnel-like passageway between the free edge of the overlapping ovale fossa valve and its muscular rim [2]. While an ASD involves a hole in the interatrial septum, the IAC has holes of different sizes connected to each other via a tortuous channel. In addition, the lengths of the open IACs in the present study were 14.9 mm and 16.1 mm. A tunnel-like PFO with a channel measuring longer than 12 mm creates complications, and standard closure procedures implemented through the PFO can be unsuccessful [25].

**Table 2:** Variants of interatrial communications.

Types	Prevalence	Sites	shape	Symptoms
IAC	6.5% of the adult embalmed cadavers	On the right surface of the interatrial septum, the left border of the interatrial septum above the fossa ovalis	Oval	
		On the left surface of the interatrial septum, the left border of the septum	Linear or round	
PFO	25% or 27.3% of the adult cadavers	Between the free edge of the overlapping ovale fossa valve and its muscular rim	A tunnel-like passageway	Stroke, migraine with aura
Atrial septal pouch	Left septal pouch: 40.8% or 90% of healthy adults cadavers or randomly selected adults cadavers	Partial fusion of the primary and secondary septum	A small, kangaroo pouch-like structure	Blood stasis, thrombus formation
	Right septal pouch: 5.1% or 5.5% of healthy adults cadavers or randomly selected adults cadavers			No clinical significance
ASDs				
• Ostium secundum	75% of ASD	Fossa ovalis	Round, oval, irregular	Atrial tachyarrythmias, Paradoxical embolism, right ventricular failure, pulmonary hypertension, right ventricular failure
• Ostium primum (Atrioventricular)	15–20% of ASD	Inferior portion of the interatrial septum resulting from abnormal fusion of the endocardial cusions		
• Sinus Venosus	5–10% of ASD	Near the superior or inferior vena caval entry		
• Coronary Sinus	Rare	A defect in the coronary sinus wall		

## Conclusions

The present study has revealed that the IAC exhibits characteristics distinct from other types of interatrial communication. The IAC seems to be a new type of interatrial communication able to cause a cryptogenic stroke or transient ischemic attack. The IAC is different from the PFO or classical ASDs (secundum or sinus venosus types). As a wide IAC can cause a right-to-left shunt, comprehension of the IAC is important. These findings of the IAC can provide useful data for evaluating its site through the interatrial septum. Furthermore, the results of the present study are expected to lead to a better understanding of the pathophysiology of the heart.
